# The role of the ubiquitin proteasome system in cerebellar development and medulloblastoma

**DOI:** 10.1186/s13041-015-0155-5

**Published:** 2015-10-17

**Authors:** Jerry Vriend, Saeid Ghavami, Hassan Marzban

**Affiliations:** Department of Human Anatomy and Cell Science, Rm129, BMSB, 745 Bannatyne Avenue, Winnipeg, MB Canada; Children’s Hospital Research Institute of Manitoba (CHRIM), College of Medicine, Faculty of Health Sciences, University of Manitoba, Winnipeg, MB R3E 0J9 Canada

**Keywords:** Cerebellar granule cells, Sonic hedgehog, WNT1, SCF-β-TrCP, E3 ligase F-box/WD repeat-containing protein 7, E3 ligase S-phase kinase-associated protein 2

## Abstract

Cerebellar granule cells precursors are derived from the upper rhombic lip and migrate tangentially independent of glia along the subpial stream pathway to form the external germinal zone. Postnatally, granule cells migrate from the external germinal zone radially through the Purkinje cell layer, guided by Bergmann glia fibers, to the internal granular cell layer.

Medulloblastomas (MBs) are the most common malignant childhood brain tumor. Many of these tumors develop from precursor cells of the embryonic rhombic lips. Four main groups of MB are recognized. The WNT group of MBs arise primarily from the lower rhombic lip and embryonic brainstem. The SHH group of MBs originate from cerebellar granule cell precursors in the external germinal zone of the embryonic cerebellum. The cellular origins of type 3 and type 4 MBs are not clear.

Several ubiquitin ligases are revealed to be significant factors in development of the cerebellum as well as in the initiation and maintenance of MBs. Proteasome dysfunction at a critical stage of development may be a major factor in determining whether progenitor cells which are destined to become granule cells differentiate normally or become MB cells. We propose the hypothesis that proteasomal activity is essential to regulate the critical transition between proliferating granule cells and differentiated granule cells and that proteasome dysfunction may lead to MB. Proteasome dysfunction could also account for various mutations in MBs resulting from deficiencies in DNA checkpoint and repair mechanisms prior to development of MBs.

Data showing a role for the ubiquitin ligases β-TrCP, FBW7, Huwe1, and SKP2 in MBs suggest the possibility of a classification of MBs based on the expression (over expression or under expression) of specific ubiquitin ligases which function as oncogenes, tumor suppressors or cell cycle regulators.

## Introduction

Cerebellar neurons can be classified into inhibitory gamma-butyric acid (GABAergic) and excitatory glutamatergic neurons [1]. The cerebellar GABAergic neurons including Purkinje cells, and inhibitory interneurons, Golgi cell, basket and stellate cells, are found in the cortex [2–4]. Glutamatergic neurons include the granule cells, unipolar brush cells, and large projection neurons in the cerebellar nuclei [4–8]. The cerebellar granule cells are the smallest and most abundant neurons in the vertebrate brain [4, 9].

Cerebellar neurogenesis begins at around E8-E13/14 from cerebellar germinal zones that can be categorized in four zones: 1. The internal germinal zone (ventricular zone), 2. The external germinal zone (external granular layer), 3. Upper rhombic lip (caudomedial germinal zone) and 4. The rostral germinal zone (mesencephalon). In this review we will discuss development of the rhombic lip and the external germinal zone, the source of granule cells, as they relate to the major types of medulloblastomas (MBs). Herein we also relate normal cerebellar development to signal transduction factors, and discuss ubiquitin-proteasome dysfunction during development as a major factor in development of MBs.

### Granule cell formation and cerebellar germinal zone

The rhombic lip is a highly proliferative region of the neural fold located in rhombencephalon (r) that can be divided into upper rhombic lip (r1) and lower rhombic lip (r2-r8) [9, 10].

The upper rhombic lip or caudomedial germinal zone of r1 is the main source of granule cell precursors which are located at the interface of the ventricular zone and the roof plate [4]. Granule cells precursors are created in the caudomedial germinal zone from E12.5 to E17 and they migrate rostrally in a subpial stream of cerebellar primordium to establish the external germinal zone, which is the location of non-committed granule cell precursors [10, 11].

The MATH1 gene, which encodes a bHLH transcription factor, is expressed in the rhombic lip as well as in proliferating granule cell precursors in the external germinal zone. Targeted disruption of this gene results in complete loss of the granule cell lineage [12, 13]. Math1 and Wntless (Wls) are expressed complementary in the exterior and interior face of the rhombic lip [10]. Barhl1 is a mouse homeobox gene that plays a role in cerebellum development. It is activated by the transcription factor MATH1, possibly in response to the growth factor BMP. In glutamatergic neurons, the developmental protein PAX6 is downstream in the MATH1 pathway [10], which includes sequential expression of TBR2, NeuroD, and TBR1 [14, 15].

Wnt1 is a glycoprotein that is important in maintaining the isthmus organizer at the junction of mesencephalon and rhombencephalon [16]. WNT1 is also expressed at the caudal end of the caudomedial zone [17]. Mutations in Wnt1 produce severe midbrain and cerebellar defects [18]. However, the distribution and molecular identity of WNT1 expressing progenitors have not been carefully described in r1. Hagan and Zervas observed that WNT1 lineage marked later in development predominantly gave rise to granule cells in the adult cerebellar cortex [17]. Lorenz et al. [19] reported that Wnt/β-catenin signaling pathway is essential in proliferation and differentiation of cerebellar granule neuron precursors. It is also been reported that lower rhombic lip progenitors express WNT1 which give rise to neurons of the precerebellar system, a major source of afferent projections to the cerebellum [17].

**Fig. 1:**
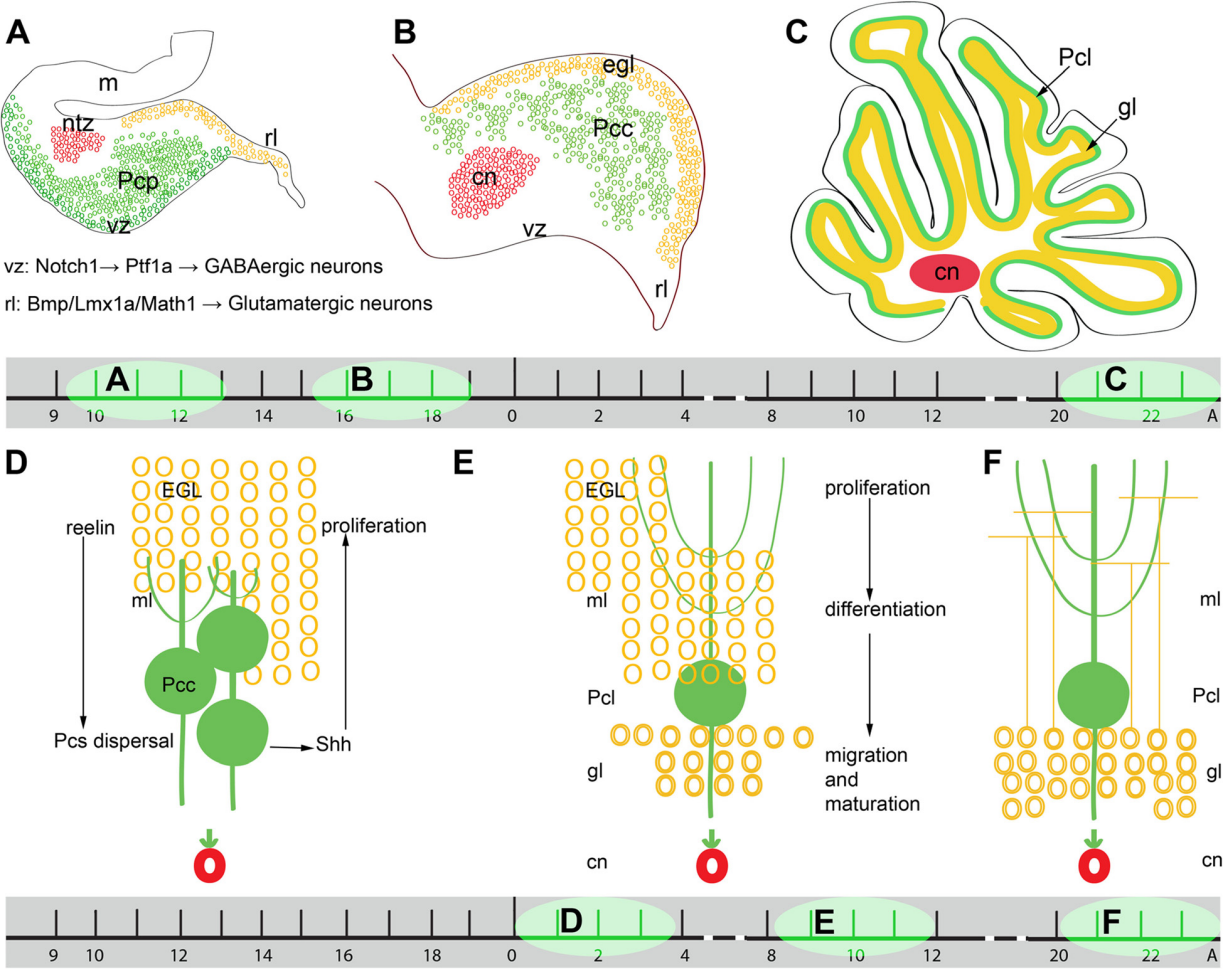
Timetable and sources of the cerebellar neurogenesis and granule cells formation: **a-c** Schematic illustration of the spatiotemporal parameters at sagittal sections of the early cerebellar development (embryonic (**e**) day 10–13 (E10-E13) (**a**), E15-E17 (**b**), and postnatal (P) day 20 (P20) (**c**). Neuroepithelium of 4^th^ ventricle (ventricular zone (vz)) is sources of all GABAergic neurons including Purkinje cells (green) under control of NOTCH1 and PTF1a pathway. Rhombic lip (rl) under influence of BMP/LMX1a develop and is sources of all glutamatergic neurons including cerebellar nuclei neurons (red) and external germinal zone (orange; source so granule cells). **d-e** Schematic illustration of the spatiotemporal parameters in corticogensis and granule cells development Purkinje cells (green) express SHH that increases proliferative activity of external germinal zone (EGZ) cells (precursor of granule cells). Reelin express from precursor of granule cells and causes dispersal of Purkinje cells cluster (**d**) to monolayer (**e-f**). **e** Granule cells differentiate and migrate cross Purkinje cells layer to final destination i.e. granular layer and granule cells development is completed by maturation in this layer. Abbreviations: Pcc = Purkinje cell clusters, Purkinje cells precursor (pcp), mesencephalon (m), rhombic lip (rl) E = embryonic day, EGL (EGZ) = external germinal layer (zone), gc = granule cells, m = mesencephalon, NTZ = nuclear transitory zone, A = Adult, pcl = Purkinje cell layer, rl = rhombic lip, ml = molecular layer

The external germinal zone is a subpial location of proliferating cerebellar granule cell precursors [4]. The post-mitotic granule cells develop axons that extend among the parallel fibers in the developing molecular layer while the somata migrate inward to the granular layer through the developing Purkinje cell layer (Fig. 1).

SHH-signaling has been shown to be an important driver of granule cell progenitor proliferation [20, 21]. SHH secreted by Purkinje cells starting at around embryonic day 18.5 in the mouse cerebellum [22]. Its receptor patched (PTCH1, alternatively referred to as PTC1, PTC, or by the family name PTCH), a protein that interacts with smoothened (SMO), and its effector, GLI-2, exhibit similar temporal patterns of immunoreactivity in the external germinal zone [22, 23].

The Bmi1 gene, which promotes cell proliferation, is expressed strongly in the cells of the external germinal zone at around E16.5 during mouse cerebellar development. Bmi1 is expressed in parallel with N-MYC and cyclin D2 in the external germinal zone of human cerebellum from 17 weeks gestation to 2 months postnatal [24]. The Bmi1 gene is described as an oncogene [25] which is transcribed as a transcription repressor which inhibits the expression of the ubiquitin E3 ligase, Fbw7 [26]. BMI1 is also considered to have a critical role in maintaining cyclin E (a substrate of Fbw7) [26].

### Types of medulloblastomas

MBs are the most common malignant childhood brain tumor. Some of these tumors develop from the progenitor cells in the external germinal zone of the embryonic cerebellum [27]. The cerebellar granule cell is the most numerous neurons in the nervous system and is considered the likely source of a subgroup of MBs [28]. Clinically and molecularly four main types of MB are recognized [29, 30]. These are described as the WNT group, the SHH group and two other groups referred to simply as groups 3 and 4. The relative percentages of the various groups of MBs are approximately 11 % (Wnt), 28 % (SHH), 38 % (Group 3) and 34 % (Group 4) [31]. Subtypes of each of the main groups have been defined based on genetic and molecular markers [32]. According to the data of Schuller et al. [33], MBs of the SHH group originate from cerebellar granule neuron precursors (CGNP). However the cellular origins of group 3 and group 4 MBs have not been definitively determined.

### The WNT group of medulloblastomas

In the WNT group of MBs, there is overexpression of genes associated with the WNT (Wingless-related integration site) signalling pathway, as well as overexpression of TGFβ [34]. Mutations of β-catenin are frequently associated with this group. According to Kool et al. [34] the TGFβ pathway may be activated in MB by β-catenin mutations.

The WNT signalling pathways have been studied for their role in embryonic development and in carcinogenesis. β-catenin is the main mediator of the classical WNT pathway. It functions as a transcription factor which ‘moonlights’ as a cell adhesion molecule [35]. β-catenin has a short half-life, which depends on phosphorylation, ubiquitination and degradation by the proteasome [36, 37]. Accumulation of unphosphorylated β-catenin facilitates translocation to the nucleus. To act as a transcription factor in the nucleus β-catenin interacts with LEF/TCF (Lymphoid enhancer factor/T cell factor [38]. Disturbances of WNT/β-catenin signalling are considered to lead to defects in differentiation of mesenchymal stem cells, developmental disorders and cancer [39]. Gibson et al. [40] reported a mouse model of WNT MB with some evidence that the tumor arose primarily from the lower rhombic lip and embryonic brainstem rather than from the upper rhombic lip and cerebellum.

### The SHH group of medulloblastomas

In the SHH signal transducing pathway when one of the hedgehog family of proteins (Indian Hh, Sonic Hh, Desert Hh) binds to the transmembrane receptor PTCH1, another transmembrane protein smoothened (SMO) is released to activate the GLI transcription factors. The GLI factors in turn stimulate the expression of target genes including oncogenes and tumor suppressor genes [41]. Blockade of the SHH pathway is reported to inhibit the growth of MB in culture [42].

In the SHH group of MBs there is overexpression of genes for proteins in the SHH signaling pathway [34]. Thus expression of PTCH and GLI genes may be upregulated. Mutations of PTCH1, PTCH2, SMO, and SUFU may occur in this group of MBs [43, 44]. Kool et al. [45] have recently characterized the frequency of these mutations at different ages. They found mutations of PTCH1 and SUFU were most frequent in infants; mutations of PTCH1 and TP53 (tumor suppressor p53) most frequent in young adults; mutations of PTCH1 and SMO in adults. PTCH1 mutant mice are used as a model for MBs of the SHH group. The SHH type of MBs in mouse models develop from granule cell precursors of the cerebellum [33], cells that normally differentiate into glutamatergic neurons. It has however been noted that pediatric and adult SHH MBs are molecularly distinct [43].

### Group 3 tumors and MYC signaling

The group 3 MB tumors are sometimes referred to as the MYC group since this transcription factor (which also regulates histone acetylation) is overexpressed in these tumors [29]. MYC (but not MYCN) amplification according to Taylor et al. is mostly, but not exclusively, limited to Group 3 tumors [29]. The cell surface receptor, C-met (a kinase found in progenitor cells), and its ligand HGF, apparently interact to stimulate MYC in this group of tumors [46]. Immunocytochemically these tumors are identified as positive for the NPR3 protein (Natriuretic peptide receptor C) [43]. Tumors in this group are also likely to overexpress genes for photoreceptor proteins, including S-antigen and opsin [29, 47]. In humans there are often disruptions of the p53 signaling pathway in these tumors [48]. Data in mice lacking p53 in external germinal zone progenitor cells, suggest that human Group 3 MB tumors appear to originate from these progenitor cells [48]. Patients with group 3 type tumors have the worst prognosis [30]. A mouse model of MYC-driven MB has been reported [49, 50]. In this model the cells of origin of the MB was a granule cell precursor or a stem cell not yet differentiated into granule cells.

### Group 4 tumors

The group 4 MB tumors are often identified immunohistochemically by the potassium channel marker KCNA1 [29], but less is known about the molecular characteristics of this group than of the other groups.

Recently an attempt has been made to classify MBs on the pattern of G-protein coupled receptor (GPCR) expression [51]. On this basis, Whittier and colleagues [51] distinguished 5 groups of MBs. A cluster of tumors in which the GPCR LGR5 (a stem cell marker) and GPR64 (an orphan receptor) is overexpressed corresponded to the WNT group of MBs, whereas a cluster of tumors in which the GPCR PTGER4 (a prostaglandin receptor) is overexpressed corresponded to the SHH group of MBs. The GPCR gene for melatonin (MTNR1A) was under expressed in the histopathological Groups 3 and 4, as well as the adenosine receptor, ADORA1. Four GPCRs were overexpressed in all MB groups compared to control tissues. Other investigators have reported overexpression of somatostatin receptors, SSR2, in MBs [52]. A recent report [53] provides evidence that the G protein subunit Gα_s_ is a tumor suppressor in the SHH MBs. G protein subunit Gα_s_ is found in granule cell precursors. Silencing of the gene for this protein was reported as sufficient to cause MB in mice.

Thus despite the heterogeneous nature of MBs, they can be classified histopathologically as well as by GPCR expression. Since GPCR signaling is regulated by the ubiquitin proteasome system [54] this suggests a role for the ubiquitin–proteasome system in classification of MBs as well. While accurate classification of MBs is essential to treatment, the heterogeneity of MBs makes treatment a challenge.

## The ubiquitin proteasome system and medulloblastoma

### The ubiquitin–proteasome system

The ubiquitin-proteasome system (UPS) has been described in many reviews (e.g., [55, 56]). Ubiquitin is added to proteins via a series of 3 enzymes, an activating enzyme, E1, a conjugating enzyme, E2, and an ubiquitin ligase, E3. Ubiquitination of proteins, among other functions, facilitates degradation of proteins by the proteasome. The proteasome is composed of two subunits, a 19S regulatory particle and a 20S core particle containing degradative enzymes. The molecular structure of the proteasome has been described in detail (e.g., [57, 58]).

Nahreini et al. [59] suggested that normal proteasome activity is essential during neuronal cell differentiation. If the ubiquitin proteasome system does not function appropriately during differentiation by controlling degradation of signaling and transcription factors, the normal fate of the cell will be altered. Thus, structural or functional defects in ubiquitin ligases or in components of the proteasome itself could alter the fate of the cell.

Several genes encoding signal transduction proteins involved in development of the cerebellum may be dysregulated or mutated in MB. These proteins include the transcription factors, β-catenin and GLI, as well as cMYC and NOTCH. All four of these proteins are regulated by SCF (An E3 ligase complex) mediated degradation by the ubiquitin-proteasome system [60]. β-catenin and GLI3 are ubiquitinated by SCF-B-TrCP, while cMYC is ubiquitinated by SCF-Fbw7 and SCF-SKP2, and NOTCH is ubiquitinated by SCF-Fbw7 (Table 1). Mutation of the β -TrCP recognition site on the gene for β-catenin, CTNNB1, has been reported to be associated with WNT tumors [61]. Mutations of the APC (adenomatous polyposis coli) gene are also found in some MBs [61]. APC is part of the β -TrCP ligase complex that ubiquitinates β-catenin prior to degradation by the proteasome. This is illustrated by Narayan and Roy [62] as it relates to colorectal cancer. While to the best of our knowledge no mutations of SKP2 in MBs have been reported, it should be noted that mutations of the β -TrCP complex would interfere with SKP2 degradation [63]. While mutations of Fbw7 have apparently not been reported in MBs, they have been well studied in T cell acute lymphocytic leukemia. In this disease cMYC, NOTCH and cyclin E tend to accumulate with Fbw7 deficiency, along with inactivation of p53 [64].

**Table 1:** Major substrates of SCF ubiquitin ligases, Fbw7, β-TrCP, and SKP2

SCF-Fbw7		
Notch	Cell proliferation signaling	[140–143]
c-Myc	Transcription factor	[140, 144, 145]
c-Jun	Kinase	[146]
Cyclin E	Cell cycle regulator	[26, 147]
Mcl-1	Differentiation protein	[148]
SREBP	Transcription factor	[149]
PGC-1a	Transcription factor involved in energy metabolism	[150]
Nrf-1	Transcription factor	[151]
TGIF	Transcriptional repressor of TGF-b	[152]
Aurora-A	Kinase and regulator of chromosome segregation	[153, 154]
SV40 T antigen	Viral oncogene	[155]
KLF5	Transcription factor	[156]
mTor	Kinase	[157]
SCF-βTrCP		
Gli2	Transcription factor	[158, 159]
GLi3	Transcription factor and mediator of SHH signaling	[160, 161]
B-catenin	Transcription factor and cell adhesion; mediator of Wnt signaling	[162–164]
IkBα	NFkB inhibitor	[163–166]
mdm2	Ubiquitin ligase for p53	[148]
Wee1	Cell cycle regulator kinase	
Cdc25a	Phosphatase in cell cycle regulation	[167]
Rest	Gene suppressor and chromosome stabilizer	[133]
Nrf-2	Transcription factor for antioxidant enzymes	[168]
Emi1	Cell cycle regulator kinase	[167]
HIV-1 Vpu	Viral pseudosubstrate	[169]
Per1/2	Circadian regulation	[170–172]
Snail	Neural crest regulation	[173]
DEPTOR	Autophagy inhibitor	[174]
Mcl-1	Differentiation protein	[175]
SCF-SKP2		
p27	Cell cycle regulator	[176, 177]
p21	Cell cycle regulator	[178, 179]
E2F	Transcription factor	[180]
Akt	Protein kinase	[181]
FOX01	Transcription factor	[182]
Brca2	DNA repair	[183]
cyclin A	Cell cycle regulator	[184]
cyclin E	Cell cycle regulator	[185]
c-myc	Transcription factor	[186]
HPV Viral E7	Viral oncogene	[187]

The cycle protein p27 is regulated by SCF-Skp2. Skp2 control of p27 may be particularly important in the SHH group of MBs [65]. The protein PTCH1, a receptor for SHH, is also regulated by the ubiquitin-proteasome system [66]. The HECT E3 ligase, Smurf regulates the degradation of PTCH1 [67]. The ubiquitin E3 ligase for the transmembrane protein, smoothened (SMO), is unknown.

The transcription factor MATH1 (ATOH1) is an important signaling factor in development of the cerebellum [10]. MATH1 positive cells in the rhombic lip of the developing cerebellum give rise to glutamatergic cells. MATH1 is protected from degradation by the ubiquitin-proteasome system by SHH signaling in granule cell progenitor of the external germinal zone [68]. According to these investigators the regulation of MATH1 is an important factor controlling the switch between proliferation and differentiation. The ubiquitin HECT E3 ligase, Huwe1, regulates the stability of MATH1. Disruption of SHH-MATH1 regulation may be a factor in the development of SHH MBs. Forget and colleagues [68] found if MATH1 was overexpressed, differentiation of granule cells was inhibited, and migration was impaired. They concluded that Huwe1-dependent degradation of Math1 by the proteasome was required for normal differentiation of granule neuron progenitor cells. They also found that BMP inhibited MATH1 expression by a posttranslational mechanism. Their work showed the importance of the ubiquitin-proteasome system in controlling levels of the transcription factor MATH1 in cerebellar granule cell progenitors. These investigators found that Huwe1 levels were decreased in mouse SHH MBs. In human SHH MBs low HUWE1 levels were associated with poor prognosis [68] HUWE1 may regulate the stability of other proteins, including the tumor suppressor p53 [69].

The tumor suppressor, SUFU (suppressor of fused), inhibits both the WNT and SHH pathways by promoting the nuclear export of β-catenin and GLI [70], an SHH stimulated transcription factor. Degradation of SUFU by the ubiquitin-proteasome system is stimulated by SHH [71]. SUFU interacts with GLI, to control cerebellar morphogenesis [72]. In Xenopus embryos SUFU is reported as a regulator of both WNT and SHH pathways [73] during neural induction and patterning. To the extent that SUFU regulates transcription factors in granule cells dysregulation of this protein has been suggested as contributing to the development of MB [70]. Mutations of SUFU have been reported in young children with MB [74].

In culture bone morphogenic proteins (BMP2 and BMP4) inhibit proliferation and induce differentiation of cerebellar granule neuron progenitors and MB cells. Genes regulating the BMPs are decreased in expression in mouse MBs [75]. Zhao et al. [75] reported that BMPs could be clinically useful inhibitors of MBs. The ubiquitin ligase Smurf1 is a major regulator of BMP signaling [76].

The two SCF ubiquitin ligases Fbw7 and β-TrCP have been studied for their roles in tumorigenesis. Lau and colleagues [77] reviewed the literature on how the ubiquitin-proteasome processing of substrates of these ligases promoted carcinogenesis. The Fbw7 gene, which encodes Fbw7, regulates a number of proteins important in tumorigenesis including c-JUN, NOTCH, cyclin E, SREBP1, mTOR, c-MYC, MCL-1, NRF-1 and the proto-oncogene DEK [78, 79]. According to Wang and colleagues [80] the E3 ligase Fbw7 is as a tumor suppressor in a variety of cancers. Davis and colleagues [79] suggested that Fbw7-associated tumorigenesis requires the dysregulation of multiple oncoproteins. Fbw7 has been reported to cooperate with β-TrCP in the ubiquitin-proteasomal degradation of the myeloid cell leukemia factor (Mcl-1) [81]. NRF1 in the nucleus is degraded by β-TrCP and Fbw7 and in the ER- associated pathway by the ligase Hrd1 [82].

Loss of Fbw7 may result in various types of cancer [79]. Indeed, it has been described as a ‘bona fida tumor suppressor’ [77]. Decreased Fbw7 would be expected to be of particular importance in MBs with MYC overexpression since it is the ubiquitin ligase for this protein. Rajagopalan and colleagues [83] implicated cyclin E as a possible mediator of Fbw7 mediated tumor initiation through its stimulation of genetic instability. Mao and colleagues [26] noted that the transcription repressor, Bmi1 stabilizes cyclin E levels in the cell by inhibiting transcription of Fbw7. It would be expected to stabilize other substrates of Fbw7 (Table 1) as well. Fbw7 is reported as decreased in some cases of MB [84].

The ubiquitin ligase β-TrCP might be expected to be of more significance in the WNT group of MBs via its proteolytic control of β-catenin. β-TrCP could also be of importance in the SHH group of MBs via its ubiquitination of the GLI3 protein [85]. It is also a ubiquitin ligase for MDM2, a regulator of the tumor suppressor p53 [77]. Furthermore, B-TrCP is a ligase for Emi1, an inhibitor of the Anaphase Promoting Complex (APC/C) [86]. APC/C, a tumor suppressor, itself is a ubiquitin ligase for casein kinase1δ in cerebellar granule precursor cells [87].

Hede et al., reported increased expression of the β-TrCP and SKP2 ubiquitin ligases in some MBs [84]. Overexpression of the ubiquitin ligase SKP2 is reported in various tumors and has been suggested to be of importance in the SHH group of tumors [65].

**Fig. 2:**
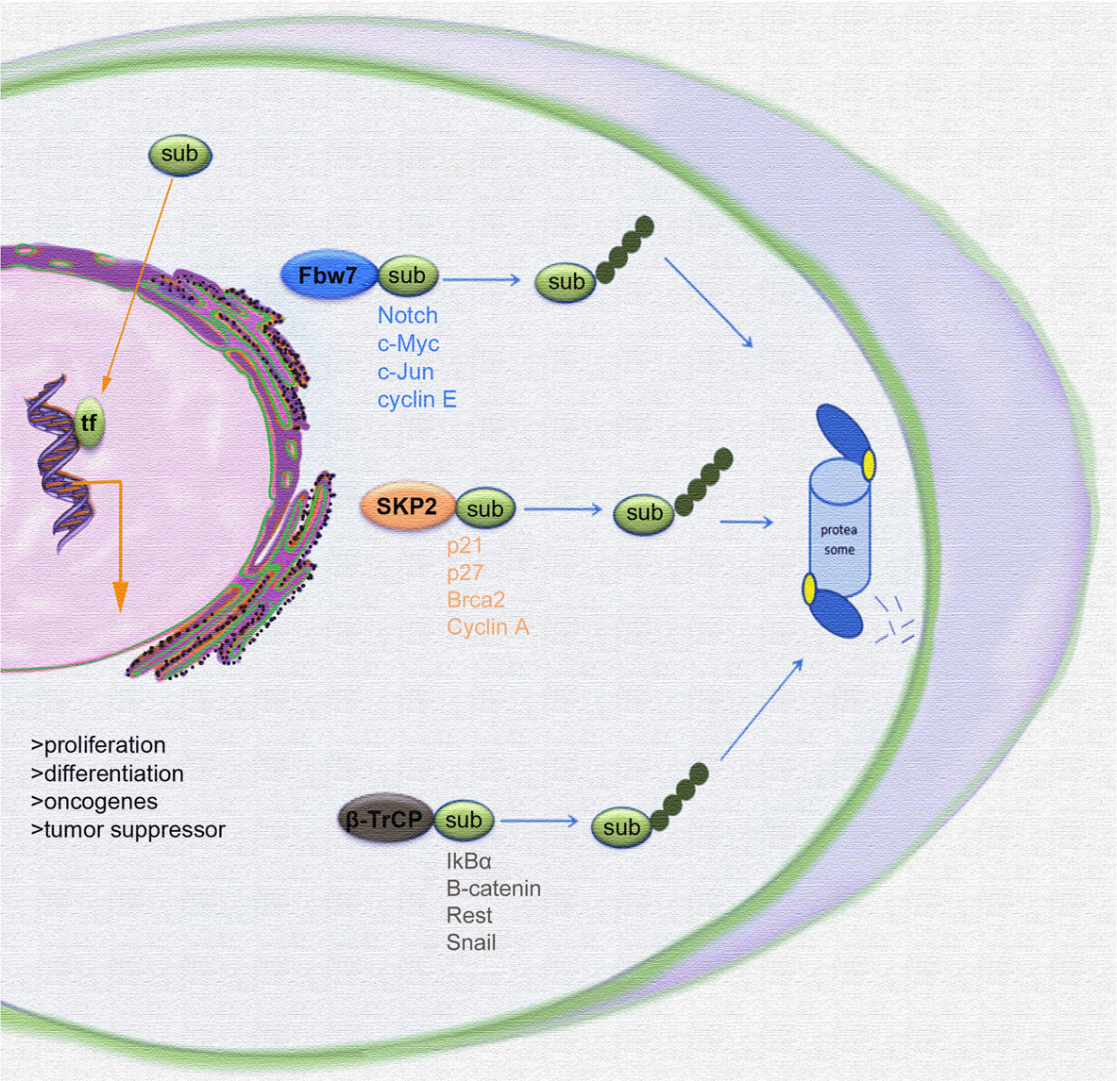
The SCF ligases, Fbw7, SKP2, and β-TrCP regulate oncogenes and tumor suppressors in medulloblastomas: Schematic illustration of the cell summarizes the important SCF ligases, Fbw7, SKP2, and β-TrCP and important substrate that after ubiquitination targeted by proteasom for degradation. Fbw7 - ubiquitin ligase F-box WD repeat containing protein 7; Skp2 - ubiquitin ligase S-phase kinase associated protein; Β-TrCP - ubiquitin ligase B-transducin repeats-containing protein.  Ubiquitin, Sub; substrate, tf; transcription factor

Dysregulation of ubiquitin ligases appears to be a component of at least some types of MB. It would be useful to have information on expression of Fbw7, SKP2, and β-TrCP in the 4 different classification types of MB. These three ubiquitin ligases have been most extensively studied for their role in initiation and maintenance of tumors [79]. They appear to be of critical significance as epigenetic factors controlling the balance of expression of oncogenes and tumor suppressive proteins (Fig. 2). They are also the three main F-box ligases (of over 70 identified) known to regulate the cell cycle [88].

### The ubiquitin-proteasome system and NF-κB in medulloblastoma

High levels of NF-κB (nuclear factor kappa-light-chain-enhancer of activated B cells) activity has been reported in MB cell lines and in MB xenograft tumors [89]. A number of drugs that inhibit NF-κB activity in cell lines inhibited tumor growth as well [89]. According to Northcott et al. [90] NF-κB signaling is particularly predominant in Group 4 MBs. The ubiquitin ligase β-TrCP regulates the degradation of the NF-κB inhibitor IkBα [91]. Proteasome inhibitors such as bortezomib are well known for their ability to inhibit NF-κB [92].

### The proteasome and DNA repair

Mutations in various genes, including those for SUFU, SMO, PTCH, MycN [45, 93], β-catenin [61], APC [94] and TP53 [95], have been reported as causal factors in MB. Mutations associated with MB suggest the possibility that DNA repair mechanisms have been compromised. One mechanism suggested as contributing to MB development is that of a defect in DNA repair mechanisms in progenitor cells [96]. Lee and colleagues [97] and Frappart et al. [98] have reported MBs arising from DNA repair deficiencies in conjunction with p53 dysfunction and genome rearrangement. One of the major functions of the ubiquitin-proteasome system is that of regulation of DNA repair [99]. Thus dysfunction of the ubiquitin-proteasome system could contribute to compromised DNA repair mechanisms leading to MBs.

### Proteasome inhibitors and medulloblastoma

A variety of signal transduction factors have been implicated in the differentiation and proliferation of MB cells (Table 1). Ubiquitination of these proteins is necessary for processing prior to degradation, or for functioning as a transcription factor. Proteasome inhibitors thus influence both gene transcription and protein degradation.

The proteasome inhibitor bortezomib, approved by the FDA for treatment of multiple myeloma and mantle cell lymphoma, has been used in preclinical studies to inhibit CNS tumor growth [100]. Bortezomib was reported to inhibit the growth of neuroblastoma cells injected into mice [100]. The signal transducing factor, TRAIL, enhanced the sensitivity of these tumors to the proteasome inhibitor [100]. Bortezomib induces apoptosis of human medulloblastoma cells and inhibits AKT and NF-κB signaling [101]. It also induces apoptosis in vivo in a mouse model of the SHH type of medulloblastoma [102]. In another mouse model of MB, in which PTCH1 is inactivated, bortezomib had anti-tumor activity, down-regulated the SHH pathway and restored PTCH1 levels [103]. Based on *in vitro* studies and on mouse xenograft models Yang et al. suggested the use of bortezomib in treatment of pediatric MBs [101]. However pharmacological studies have shown that bortezomib does not readily cross the blood–brain barrier [104]. It could however be tested in those MBs that during growth disrupt the blood brain barrier. The need for developing proteasome inhibitors that do cross the blood–brain barrier is warranted. Bortezomib can stabilize β-catenin in mesenchymal stem cells and influence their differentiation [105]. Luchetti et al. [106] suggested that at least one signaling pathway may acts as a molecular switch in differentiation of stem cells into neural progenitor cells. The ubiquitin ligase Smurf2 has been reported to enhance neuron differentiation from mesenchymal stem cells [107]. A previous review summarized the role of the ubiquitin proteasome system in differentiation of stem cells and progenitor cells [108] The role of proteasome inhibitors such as bortezomib on differentiation suggests that the UPS may have a critical role regulating the differentiation of neural progenitor cells which give rise to granule cells during the critical period when they are susceptible to being transformed into MB cells. Recent evidence indicates that the ubiquitin ligase APC/C contributes to regulation of cerebellar granule cell progenitor cells [87].

### Proteasome and apoptosis in granule cells

The proteasome has some paradoxical effects on apoptosis. The ubiquitin-proteasome system regulates several proteins related to apoptosis. In HL60 leukemia cells proteasome inhibitors activate apoptosis [109]. In cells such as sympathetic neurons, however, proteasome inhibitors may be pro-apoptotic [110]. Canu et al. reported anti-apoptotic effects in cerebellar granule cells from 8 day old rats [111]. Thus the proteasome inhibitors, lactacystin and MG132, were found to protect granule cells from apoptosis induced by decreasing extracellular potassium, if administered at the beginning of apoptosis [111]. In addition, Canu et al. [111] noted that ubiquitinated proteins accumulated in dying cells undergoing apoptosis. These findings lead Canu et al. to conclude that the cerebellar granule cell was a useful model for studying the relationship of the proteasome to programmed cell death [111]. Other investigators have noted that anti-apoptotic factors, such as Bcl-2, are overexpressed in a substantial percentage of desmoplastic MBs (SHH group) [112]. The SHH type of MB is thought to originate from cells in the external germinal layer of the cerebellum [113]. Bcl-2 expression in early differentiating granule cells (from 5 day old rats) is reported to be required for the anti-apoptotic action of the thyroid hormone, T3 [114] and cell survival. Bcl-2 is considered to play a role in regulating developmental cell death in embryonic precursor cells [114, 115]. It appears to be well expressed in undifferentiated granule cell precursors [112]. It should be noted that the protein complex Keap1 Cul3-Rbx1 is reported to be the ubiquitin ligase for Bcl-2 [116, 117]. It is also a sensor for oxidative stress [118] and is thought to act as a tumor suppressor [116].

Bobba et al. [119] reported that proteasome inhibitors prevent cytochrome C release during apoptosis in cerebellar granule neurons of 7 day old rats. They reasoned that the proteasome was required to initiate the process of apoptosis and concluded that proteasome inhibitors rescued cells from apoptosis. However, Porcile et al. [120] also using cerebellar granule neurons of 7 day old rats reported that proteasome inhibitors induce cerebellar granule cell death. These conflicting results were somewhat clarified by the results of Butts et al. [121]. They reported a biphasic effect of proteasome inhibition in 7–8 day rat cerebellar granule cells. Short term proteasome inhibition protected cells from apoptosis whereas long term inhibition was pro-apoptotic and possibly toxic [119]. Acute exposure of granule cells to proteasome inhibition increased levels of the pro-survival factor, MEF2D. Chronic exposure of granule cells to proteasome inhibition was found to increase levels of c-JUN and of the pro-apoptotic factor Bim.

It should be noted that the proteasome contributes to the degradation of anti-apoptotic proteins including Bcl-2 and IAP [122] as well as pro-apoptotic proteins including BAX [123]. The time of exposure and dose of proteasome inhibitors used can be factors in determining whether a proteasome inhibitor is pro-apoptotic or pro-survival [121, 124]. In cancer cells proteasome inhibitors tend to be pro-apoptotic [125].

Proteasome activity, however, is essential for proliferating cells. NF-κB reportedly contributes to the survival of cerebellar granule cells [126]. Its degradation is regulated by the ubiquitin-proteasome system.

### The proteasome and differentiation

Nahreini et al. [59] have studied the role of the proteasome in differentiation of mouse neuroblastoma cells. In the NBP2 cell line elevation of intracellular cAMP stimulates differentiation of these neurons. Their data showed that N-MYC and cyclin B1 transcription (and translation into protein) were decreased by cAMP-inducing agents during differentiation to the mature cell type. The proteasome inhibitor, lactacystin, also decreased N-MYC and cyclin B1 transcription into mRNA but increased the cellular concentrations of these differentiation proteins and led to cell death. They concluded that the proteasome was essential for differentiation and that the NBP2 cell model would be useful for testing a combination of cAMP induced differentiation in combination with proteasome inhibition as a treatment for neuroblastoma.

N-MYC regulates the cell cycle in cerebellar granule cell precursors [127]. SHH proliferative activity requires N-MYC expression. It is blocked by bone morphogenetic protein 2, BMP2 [128]. According to Alvarez-Rodriguez et al. [128], BMP2 promotes differentiation of cerebellar granule cell precursors. It should be noted that the ubiquitin ligase for N-MYC is HUWE1 [127].

### REST, MAD and cell cycle control

It has been reported that abnormal expression of the repressor element silencing transcription factor (REST) is one cause for MBs [129–131]. The fact that a number of MBs overexpress this factor supports this interpretation [132]. Negrini et al. [131] describe REST as an oncogene in MBs and neuroblastomas. Westbrook et al. [133] reported that the E3 ligase that regulates REST ubiquitination and degradation is β-TrCP. These investigators found that REST over-expression caused oncogenic transformation of human mammary epithelial cells. Frescas and Pagano [134] noted that REST overexpression can not only inhibit differentiation but also cause chromosomal instability, both of which can contribute to development of tumors.

Another protein expressed in the external germinal zone is the MAD3 protein, a member of a family of transcriptional regulators, to which MYC also belongs. This protein is reported to be an important regulator of granule cell precursors [135]. Both MAD2 and MAD3 are components of the spindle checkpoint of the cell cycle and inhibitors of the APC/C (anaphase-promoting complex) ubiquitin ligase [136]. APC/C in turn is regulated by β-TrCP, through degradation of the APC/C inhibitor EMI1 (early mitotic inhibitor) [86]. β-TrCP, as noted above, is also a ligase for REST, and an inhibitor of MAD expression during the spindle checkpoint of the cell cycle [137] and securin, another spindle checkpoint protein [138]. Guardavaccaro and colleagues [137] pointed out that prevention of REST degradation by β -TrCP may contribute to tumor formation by causing genomic instability.

### Perspectives of proteasome therapy in medulloblastoma

It has been suggested that proteasome inhibitors have potential as therapeutic agents in treating MBs [101]. As noted above a problem using some proteasome inhibitors to treat MBs is that the blood brain barrier limits the use of those that cannot cross it. Another major problem using proteasome inhibitors to treat MBs is their lack of specificity. Proteasome inhibitors may inhibit the degradation of both tumor suppressors and oncoproteins [139]. Inhibitors of ligases, such as inhibitors of the SKP2 ligase, would be more specific. Finally deubiquitinase inhibitors could also be useful in counteracting excess degradation of tumor suppressor proteins. Agents such as these need to be developed and tested for their effectiveness in individuals of the four main groups of MBs.

## Conclusion

Hypothesis: Proteasomal activity is essential to regulate the critical transition between proliferating granule cells and differentiated granule cells. Proteasomal dysfunction may be a major factor in determining whether progenitor cells which are destined to become granule cells, differentiate normally or instead become MB cells. Of particular significance for the development of MBs are the three SCF ligases, SKP2, FBW7, and β-TrCP, all of which are intimately involved in cell cycle regulation. Targeting SCF ligases may be effective in modulating stem cells involved in initiation of MB tumor cells. Targeting SCF ligases may also be significant for a variety of neural and non-neural tumors in which these ligases play a role in tumor initiation and in tumor cell proliferation.

### Highlights

- Several ubiquitin ligases important in development of the cerebellum are also significant factors in the development of medulloblastomas
- The ubiquitin ligase SCF-β-TrCP regulates degradation of several proteins in the Wnt and SHH group of medulloblastomas including β-catenin, Gli2/3, and mdm2
- SCF- β-TrCP, also regulates the inhibitor of NF-κB, IkBα, the transcription repressor element REST, and Emi1(an inhibitor of APC/C)
- The ubiquitin ligase SCF-Fbw7 which acts as a tumor suppressor regulates degradation of proteins of significance in type 3 medulloblastomas including Myc, Notch, cyclin E
- The ligase SCF-Skp2 which regulates the cell cycle protein p21 and p27 is elevated in a population of medulloblastomas
- The ubiquitin ligase, Huwe1, regulates degradation of Math1, which regulates the balance between proliferation and differentiation in granule neural progenitor cells

## Abbreviation

APC/C: Anaphase-promoting complex
BAX: Bcl-2-associated X protein
BCL-2: B-cell lymphoma 2
bHLH: Basic-helix-loop-helix family of proteins
BMP: Bone morphogenic protein
CGNP: Cerebellar granule neuron precursor
E1: Ubiquitin activating enzyme
E2: Ubiquitin conjugating enzyme
E3: Ubiquitin ligase
EGZ: External granular zone
GABA: Gamma-butyric acid
GLI: Glioblastoma derived transcription factor
GPCR: G-protein coupled receptor
IAP: Inhibitor of apoptosis
LEF/TCF: Lymphoid enhancer factor/T cell factor
MAD: Mitotic arrest deficient
MATH1: Protein atonal homolog 1 (alias ATOH1)
MB: Medulloblastoma
MYC: Myelocytomatosis oncogene
MCL -1: Myeloid leukemia cell differentiation protein
NF-κB: Nuclear factor kappa-light-chain-enhancer of activated B cells)
PTCH: Patched, a family of membrane receptors including PTCH1 and PTCH2
REST: Repressor element silencing transcription factor
SCF: Skp, Cullin, F-box complex
SCF- β -TrCP: E3 ligase β-transducin repeats-containing protein
SCF-Skp2: E3 ligase S-phase kinase-associated protein 2
SCF-Fbw7: E3 ligase F-box/WD repeat-containing protein 7
SHH: Sonic hedgehog
SMO: Smoothened
SUFU: Suppressor of fused homolog
TRAIL: TNF-related apoptosis-inducing ligand
Wls: Wntless
Wnt: Wingless-related integration site gene
